# PrecisePrimer: an easy-to-use web server for designing PCR primers for DNA library cloning and DNA shuffling

**DOI:** 10.1093/nar/gku393

**Published:** 2014-05-14

**Authors:** Cyrille Pauthenier, Jean-Loup Faulon

**Affiliations:** Institute of System and Synthetic Biology, Université d’Évry val d’Éssonnes, Bt. Geneavenir 6 Genopole Campus 1, 5 rue Henry Desbruères 91000, Évry, France

## Abstract

PrecisePrimer is a web-based primer design software made to assist experimentalists in any repetitive primer design task such as preparing, cloning and shuffling DNA libraries. Unlike other popular primer design tools, it is conceived to generate primer libraries with popular PCR polymerase buffers proposed as pre-set options. PrecisePrimer is also meant to design primers in batches, such as for DNA libraries creation of DNA shuffling experiments and to have the simplest interface possible. It integrates the most up-to-date melting temperature algorithms validated with experimental data, and cross validated with other computational tools. We generated a library of primers for the extraction and cloning of 61 genes from yeast DNA genomic extract using default parameters. All primer pairs efficiently amplified their target without any optimization of the PCR conditions.

## INTRODUCTION

Polymerase Chain Reaction (PCR) combined with molecular cloning has become one of the keystones in biology today. Deoxyribonucleic acid (DNA) constructions are especially important in systems biology to study the properties and interactions of molecular components in a cell. The technique is also a prerequisite for the entire field of synthetic biology in order to create new functions or chemicals in living organisms.

In the 1990s, molecular cloning was a tedious procedure. PCR was something difficult to perform because polymerases had not been much optimized yet and custom primers were expensive. In this context, the very popular Primer3 program (1) was developed to help the experimentalist in finding the best and shortest primers with the good annealing temperature and Guanine/Cytosine (GC) content to amplify a gene of interest. The algorithms from this software have been implemented in many other primer design tools dedicated to specific tasks such as designing primers in batches (2) designing specific primer pairs at genome scale (3–5), designing probes for real-time PCR (6–8) and detecting splicing variants (9–11) or Single Nucleotide Polymorphisms (SNPs) (12,13) in different organisms. Similarly, a few private companies developed primer library design tools, such as Illumina DesignStudio (14), Agilent SureDesign (15) and Life Technologies Ion AmpliSeq Designer (16) for next generation sequencing target enrichment.

Starting from the 2000s, the development of high-fidelity polymerases combined with high-performance cloning techniques began to change the practices in molecular biology. On one hand, academic researchers and companies started to build large libraries of DNA constructs manually or using liquid handling platforms. On the other hand, a deeper understanding of regulation sequences and protein structure pushed molecular and synthetic biologists to devise constructions with a single-nucleotide precision. The development of Gibson (17) and GoldenGate (18) assembly techniques made scar-less DNA construction possible in a high-throughput fashion. It is now possible to make enzyme libraries for screening fusion protein libraries with no artificial amino-acid insertion or to perform large scale DNA shuffling experiments (19).

Few Computer-Aided Design (CAD) tools for synthetic biology integrate primer design routines. One may mention Gibthon (20) and Geneious RC7 (21) for Gibson assembly, Maestro (22) for GoldenGate assembly or j5 (23) (commercial version teselagen (24)) for Gibson, GoldenGate, Mock and LIC. All of these tools, however, do not offer a primer designer integrating a proper melting temperature calculator capable of handling new generation high-fidelity polymerases except for j5. Single-nucleotide precision cloning poses new constraints for primer design and leaves little room for primer sequence optimization, some work has been done to incorporate position constraints in the Primer3 software in a new version called Primer3Plus (25) with the function ‘pick_cloning_primers’. However, this software sometimes refuses to design the primer at the desired position unless the constraints are completely released. Hillson *et al*. (23) noticed this problem and implemented an algorithm in j5 to progressively release the constraints of Primer3 each time it refuses to design the desired primer.

There is thus a need for a new primer design algorithm meant to directly match the constraints of modern cloning techniques, not relying on Primer3 which was conceived for other purposes. We entirely reconsidered the primer design problem trying to identify the needs of the users and the rules that increase the performances of the primers carrying 5′-extensions, as these extensions are required for most DNA assembly techniques. We also implemented the most up-to-date melting temperature methods validated on experimental data and proposed a modification in the thermodynamic constant formula, to make it more relevant in the context of PCR extraction from genomic DNA template.

In our quest for having the simplest user interface possible, we implemented many widely used new generation polymerase buffers as pre-set option, as the ionic content is not always known in the literature. Finally, we decided to give the users a complete freedom on the sequence of the primer's 5′-extensions because we believe experimentalists prefer to design them precisely themselves rather than being constrained by an interface such as a CAD tool. A comparison of PrecisePrimer features with those of other existing tools is given in Table 1.

**Table 1.** Comparison with other existing primer design tools available

	Up-to-date melting calc.	Design batches	Position constraints	Batch cloning extension	Pre-set buffers
Primer3 (1)	X			*	
BatchPrimer3 (2)	X	X		*	
Primer3Plus (25)	X		X	*	
Gibthon (20)			X	Gibson	
Maestro (22)			X	GoldenGate	
Geneious RC7 (21)		X	X	Gibson	
J5/TeselaGen (23,24)	X	X	X	Any**	
PrecisePrimer	X	X	X	Any***	X

^*^The cloning extensions can be added manually afterwards.

^**^J5 can automatically generate cloning extensions for Gibson, GoldenGate and Mock. By declaring the desired overhang as distinct parts it is possible to get custom overhangs included in the primer.

^***^PrecisePrimer gives the complete freedom to design any extension on both sides of the amplicon.

## MAIN ASPECTS OF THE PRIMER DESIGNER

### Parameters and interface

In order to amplify a segment of DNA with single-nucleotide precision, such as a gene starting from the start codon to the stop codon, the only degree of freedom left for primer optimization is how much of the interior sequence of the DNA template is taken as a part of the primer. Such primers may not be optimal from the point of view of length and GC content but new generation polymerases are still capable of working efficiently on such primers, as it is generally known by experimentalists and demonstrated in this article.

When generating a library, it is easier for all the primers to have the same melting temperature in order to amplify all genes in the same PCR tube strip or plate. PrecisePrimer first asks for the optimal melting temperature desired by the user and the melting temperature tolerance windows in which the user considers the primer to be valid. Standard PCR protocols often recommend to make an annealing period 5°C below the targeted melting temperature. We therefore propose to set the tolerance window value at ±5°C.

It is also generally recommended to have the 3′ end of the primer finishing by a G or a C to increase the stability of the primer-template dimer and ease the initiation of polymerase replication. The users have the choice between forcing the primer to finish by a G/C or to have the annealing temperature as close as possible to the optimal melting temperature provided.

The program asks for the sequence of the 5′-extension to add on the forward and reverse primer. These 5′-extensions can be used for cloning using any classical or new generation cloning method such as Gibson (17) or GoldenGate (18) or just to stabilize the PCR amplicon adding G/C to prevent dangling-end formation.

In order to calculate accurately the melting temperature, the users have to specify which buffer is used for the amplification. They can choose between using pre-set polymerase buffers or to specify the ionic content of the buffer and the algorithms they want using the custom-buffer mode.

The library of sequences to amplify should be provided in the form of a FASTA file. The software returns a result page in which all primers are shown and can be downloaded as a FASTA or a CSV file. The validation plot showing the performance of the melting temperature algorithm used on experimental data and the logs of the program are also provided.

### Primer design algorithm

**Figure 1.**
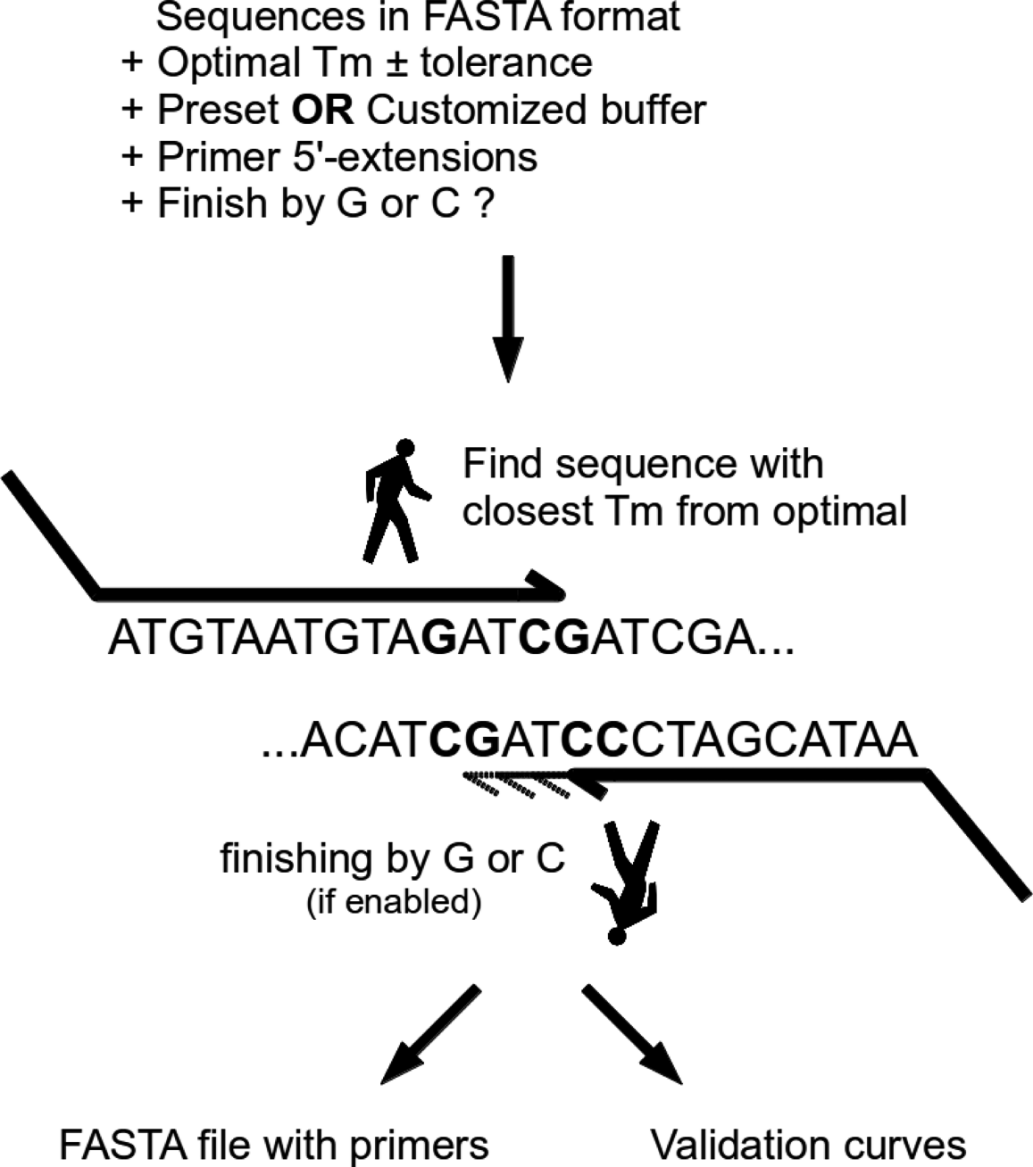
Schematic of the PrecisePrimer algorithm. The program walks from both ends of the sequence and stores all possible primers having a melting temperature comprize within the window defined by the optimal melting temperature ± tolerance. Then, it sorts all possible primers and picks the best one according to the constraints. Finally, it adds the flanking 5′-extension provided to the forward and reverse primer and returns the output files.

All the parameters described in the previous section are fed into the PrecisePrimer program. The primer design algorithm walks from the beginning and the end of each provided DNA sequences and analyses all the primer possibilities inside the tolerance window centred on the specified optimal melting temperature. Then, the program ranks the solutions and chooses the best primer pairs according to the constraints. It finally returns the pair of optimal primers, flanked by the 5′-extensions provided by the user. The program work-flow and algorithm are illustrated in Figure 1.

### Melting temperature and polymerase buffers

A trustworthy primer designer is required to have an accurate melting temperature calculator. In this work, we particularly insisted on validating the melting temperature algorithms implemented in our software. We used the best calculation methods available in the literature based on the benchmark made by Wittwer *et al*. (26). The performances of our calculator were benchmarked against experimental melting temperature values taken from the supplementary materials of the same article.

Large scale library building often requires to use high fidelity polymerases. For most of them however, the buffer composition is proprietary and the exact ionic composition is unknown to the final user. This is a problem for any primer design tool, because the composition in monovalent and divalent ions is crucial to predict the melting temperature of the primer accurately.

We obtained the monovalent equivalent concentration for all ions present in the New England Biolab (NEB) polymerase buffers when using the standard protocol provided with the enzyme. We introduced these values in our calculator and showed that it returns the same melting temperatures as the NEB calculator available online (supplementary material). Since this calculator is widely used by the biology community for manually designing primers, we believe that reproducing these results may increase the confidence final users may have in our tool.

### Discussion on melting temperature thermodynamics in the context of tailed PCR primers

Our program implements the popular nearest-neighbour thermodynamic-based algorithm using thermodynamic parameters from Breslauer (27) and SantaLucia (28) and one empirical formula coming from Wittwer *et al*.’s work (26). All the details about the melting temperature calculation formula and parameters used are provided in the documentation of the program and supplementary materials, Section S1 and S2.

By reviewing the literature and the code of open-source melting temperature calculator available on the internet we noticed that thermodynamic constant *K* is calculated using a formula based on a hypothesis that does not seem relevant in the context of a PCR with 5′-extensions. Historically, the thermodynamic parameters Δ*H* and Δ*S* for nearest-neighbour nucleotide pairs were measured on dumbbell-like structure or in a situation in which there was the same amount for the two DNA probes (28). In this case the equilibrium thermodynamic constant *K* is expressed as follows:

(1) \begin{equation*} K = \frac{4}{[{\rm Primer}]} \end{equation*}

where [Primer] is the concentration of primer used in the mixture. This formula is implemented in most calculators available online. However, in the beginning of the PCR reaction, which is the most crucial moment for the success of the amplification, the primer is in large excess compared to the template. In these conditions, the thermodynamic constant is expressed as explained by Wittwer *et al*. (26).

(2) \begin{equation*} \frac{1}{K} = [{\rm Primer}] - \frac{[{\rm Template}]}{2} \end{equation*}

where [Template] is the concentration of the template DNA. In the very beginning of the PCR, the template concentration is generally three orders of magnitude below that of the primer, and therefore the formula simplifies as (3).

(3) \begin{equation*} K = \frac{1}{[{\rm Primer}]} \end{equation*}

This formula is more relevant in the context of the PCR than the commonly used formula (1) and the annealing temperature calculated with the latter has an approximate two degrees of difference compared to (2). Moreover, in the case of PCR primers with 5′-extensions, the melting temperature of the full primer annealed with the amplicon is much higher than just the melting temperature of the part of the primer annealed with the template. According to our hypothesis, (3) is more relevant to the problem. In order to keep the compatibility with the NEB calculator, we use (1) for the calculation with NEB pre-set buffer, however, in the custom mode, we propose (3) by default.

## COMPUTATIONAL VALIDATION

**Figure 2.**
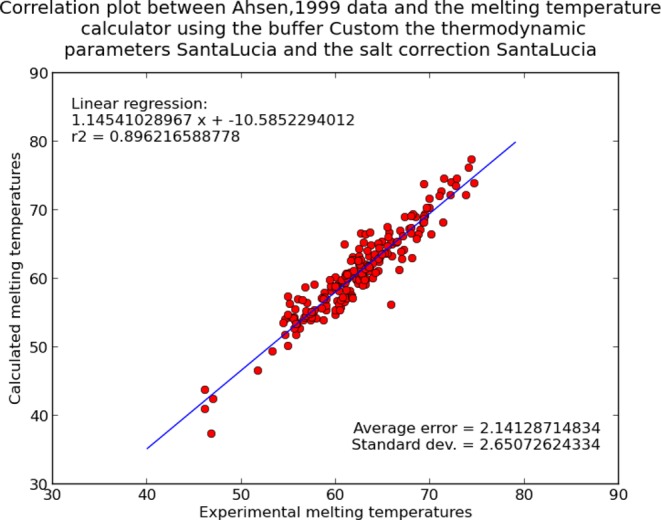
Correlation between the experimental melting temperature values from (26) and those obtained with our melting temperature algorithm using SantaLucia thermodynamic parameters (28), the SantaLucia salt correction (28) with Wittwer *et al*. parameters (26) and the (9) thermodynamic constant.

Our different thermodynamic tables and salt correction formulas have been validated by comparing predicted melting temperature with experimental melting temperature results collected from the supplementary material of an earlier paper (26), on an XY scatter plot, and calculated the linear regression. All combinations of algorithms were systematically assayed. The validation plots and parameters of the regression are shown in the supplementary materials (Figures S1–S3) and on the result page of the program. The validation curve for the best combination of parameters is shown in Figure 2. These parameters are proposed as default when using the custom buffer mode. The validation graph of the chosen melting temperature algorithm is shown to the user on the result page.

## EXPERIMENTAL VALIDATION

We proceeded to an experimental validation of our PCR design methodology by amplifying 61 fragments of genes from 1 μg *Saccharomyces cerevisiae* genomic extract using the NEB high-fidelity Q5 polymerase.

The primers were generated using PrecisePrimer configured with the pre-set Q5 polymerase buffer, and a target annealing temperature of 60°C. The oligo-nucleotides were synthesized by MWG-Eurofins with standard desalting purification and shipped in the form of a 96 well-plate. Primer sequences are available in Table S1 of the supplementary materials. The PCR was performed with 55°C annealing temperature, in the standard conditions of the enzyme protocol (30 s/longest amplicon size in kilobases) and 30 amplification cycles.

Out of the 61 gene fragments amplified, 59 showed the expected size on gel in the first PCR round (Figure S4–a). In order to compensate for pipetting errors or any other factors that may have affected the PCR, we repeated the PCR of the two remaining fragments in the same conditions the following day. The two remaining DNA fragments amplified successfully the second time (Figure S4-b). The results presented here were results we obtained at the first try, and no optimization of the PCR conditions had been made prior running the experiment shown in the paper. More experimental details are provided in the section S4 of the supplementary materials.

Overall, using the default parameters of the software, we obtained efficient amplification for all the designed primer pairs without any optimisation of the PCR conditions (see Figure S4). This demonstrates the simplicity and the reliability of our primer design methodology and our software.

## CONCLUSION

PrecisePrimer is the first easy-to-use and experimentally verified batch primer design software offering simultaneously single-nucleotide precision, custom overhangs and preset buffers. It can be used for any restriction-digest, GoldenGate, Gibson or Ligation Independent Cloning (LIC) with the flexibility of a manual primer design and the possibility to deal with batches of sequences. The melting temperature calculation has been given particular attention to be compatible with high-fidelity polymerases, to comply accurately with experimental melting temperature data and to have improved thermodynamics in the context of PCR extraction on genomic DNA template. The performance of this primer designer software has been demonstrated experimentally by amplifying 61 DNA fragments from yeast genomic extract. All of them were efficiently amplified with no optimization of PCR conditions. We believe our tool will help experimentalists to quickly design high-throughput DNA cloning or shuffling experiments with single-nucleotide precision in the future.

## AVAILABILITY & LICENCE

The webserver is available at: https://absynth.issb.genopole.fr/PrecisePrimer. The source code is not available and its usage is restricted to the terms of use available on the dedicated page.

## SUPPLEMENTARY DATA

Supplementary Data are available at NAR Online.

Supplementary Data
