# Supplementary material for: PrecisePrimer: an easy-to-use web server for designing PCR primers for DNA library cloning and DNA shuffling
Source: Nucleic Acids Res. 2014 May 14;42(Web Server issue):W205–9. doi: 10.1093/nar/gku393 (PMC4086104; doi:10.1093/nar/gku393)
Supplement: Supplementary Data [file supp_42_W1_W205__index.html]

Supplementary Data 

# PrecisePrimer: an easy-to-use web server for designing PCR primers for DNA library cloning and DNA shuffling

## Supplementary Data

**Files in this Data Supplement:**

- SUPPLEMENTARY DATA
