# Supplementary material for: PrecisePrimer: an easy-to-use web server for designing PCR primers for DNA library cloning and DNA shuffling
Source: Nucleic Acids Res. 2014 May 14;42(Web Server issue):W205–9. doi: 10.1093/nar/gku393 (PMC4086104; doi:10.1093/nar/gku393)
Supplement: Supplementary Data [file supp_gku393_nar-00273-web-b-2014-File008.pdf]

PrecisePrimer: an easy to use webserver for designing PCR primers for DNA libraries cloning and DNA Shuffling

Cyrille Pauthenier, Jean-Loup Faulon, institute of System and Synthetic Biology
University of Évry-Val-d'Essonne – CNRS FRE3561
Genopole Campus 1, Genavenir 6, 5 rue Henri Desbruères
91030 Evry Cedex, France

Supplementary materials

S1 Melting temperature calculation

Our program implements the popular nearest neighbour thermodynamic based algorithm using thermodynamic parameters from Breslauer [1] and SantaLucia [2].

$$\Delta H = \Delta H_{init} + \Delta H_{term} + \sum_{NN} \Delta H_{NN} \tag{1}$$

$$\Delta S = \Delta S_{init} + \Delta S_{term} + \sum_{NN} \Delta S_{NN} \tag{2}$$

Where ΔH and ΔS are respectively the free enthalpy and the entropy of the primer-template dimer, ΔH_{init} ΔS_{init} respectively the initiation enthalpy and entropy, ΔH_{term} and ΔS_{term} the termination enthalpy and entropy of the primer dimer. NN is the nucleotide pair in the primer-template dimer, ΔH_{NN} and ΔS_{NN} the corresponding enthalpy and entropy. The melting temperature is given by classical thermodynamics (3) [2].

$$Tm^0 = \frac{\Delta H}{\Delta S + R.log(K)} \tag{3}$$

Where R is the universal gas constant and K is the equilibrium thermodynamic constant. Tm^0 is the melting temperature in Kelvin at 1 M NaCl concentration.

S2 Salt correction

We implemented three different salt correction methods from SantaLucia [2] (4), Schilkraut [3] (5) and Owczarzy [4] (6).

$$\Delta S = \Delta S^0 + 0.847.(length - 1).log([MonoEq]) \tag{4}$$

$$SCorr = 16.6.log_{10}([MonoEq]) \tag{5}$$

$$Tm = Tm^0 + SCorr - 273.15$$

$$SCorr = (4.29.GC - 3.95).10^{-5}.log([MonoEq]) + 9.4.10^{-6}.log([MonoEq])^2; \tag{6}$$

$$Tm = \frac{1}{\frac{1}{Tm^0} + SCorr} - 273.15$$

Where ΔS^O is the entropy given by the thermodynamic tables, $length$ the length of the primer-template dimer, $SCorr$ the salt correction and GC the GC content of the primer-template dimer. The melting temperature Tm is expressed in Celsius. $[MonoEq]$ is the monovalent ion equivalent from Wittwer & co-workers [5] (7).

$$[MonoEq] = \sum[Monoval] + 120\sqrt{\sum[Dival] - [dNTPs]} \tag{7}$$

Where $[Monoval]$, $[Dival]$ and $[dNTPs]$ are the concentrations of the monovalent ions, divalent ions and dNTPs respectively. The post calculation adjustment for DMSO was taken from this paper [6] (8).

$$Tm^{final} = Tm - 0.6.\%DMSO \tag{8}$$

Where $\%DMSO$ is the percentage of DMSO in the solution, and Tm^{final} the final melting temperature in Celsius.

S3 Validation graphs

The different combination of algorithms were assayed on experimental data from this article [5]. The results are shown on Figure S1.

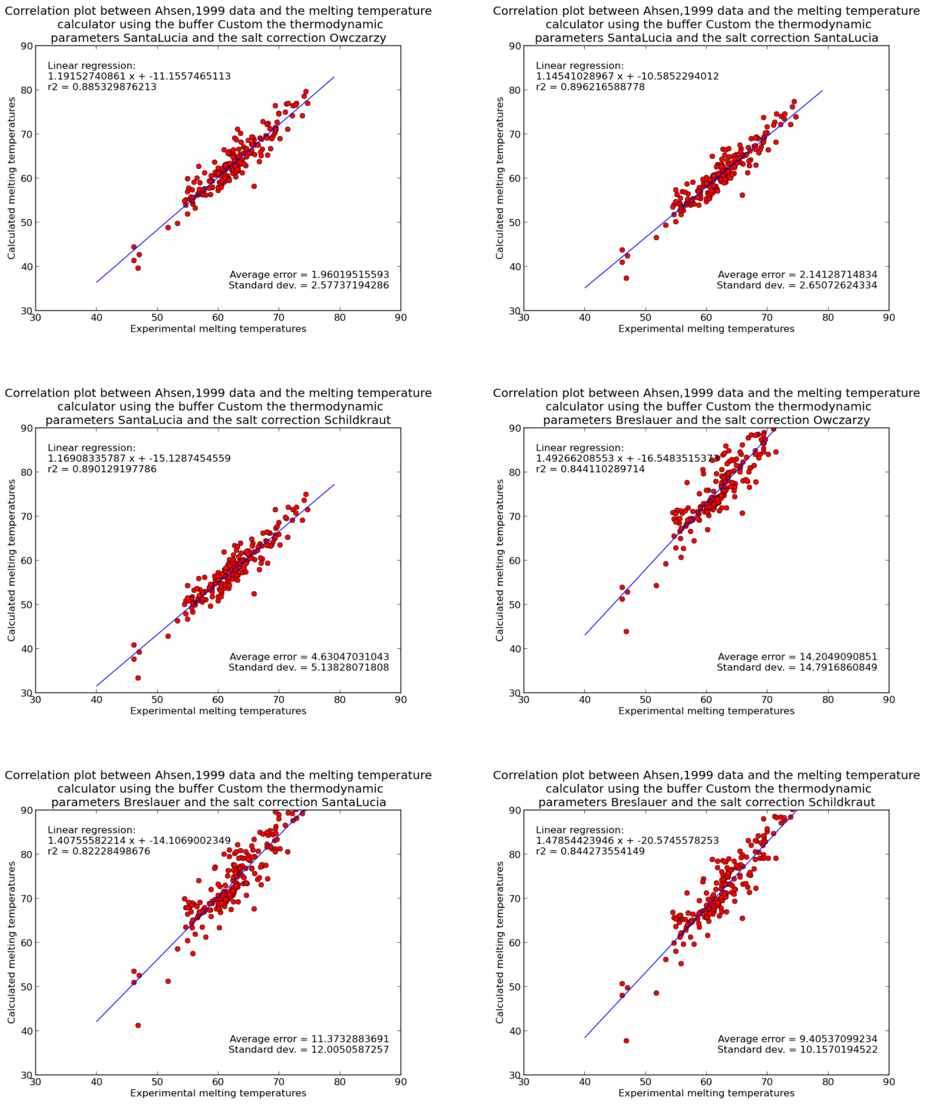

Figure S1: Validation curve of the algorithms on experimental data, with the 6 combinations of algorithms proposed.

The algorithms proposed are capable of reproducting the NEB melting temperature calculation. The set here were random sequences calculated with the NEB calculator available on-line. The melting temperature calculation is compared to the one provided by our calculator. There are only two plots because the algorithms either use SantaLucia [2] thermodynamic table with Owczarzy [4] salt correction for all the polymerases but for the Phusion polymerase which uses Breslauer thermodynamic table [1] and Schilkraut [3] salt correction. The results are shown on Figure S2.

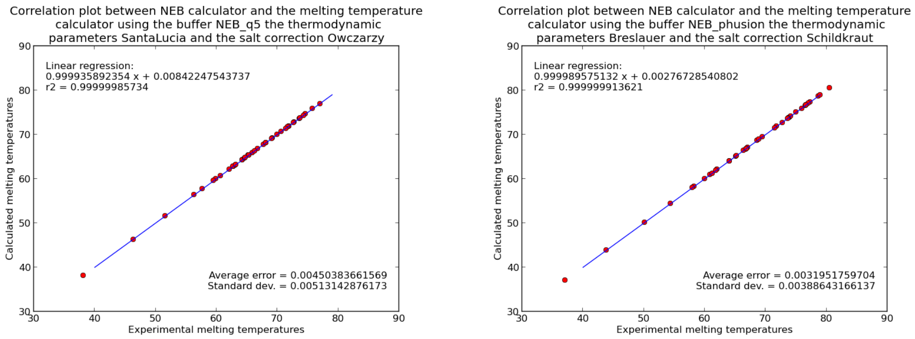

Figure S2: Validation curve of the algorithms on random sequences calculated with NEB tool compared with PrecisePrimer algorithms.

The empirical formula from this paper [5] was also implemented in PrecisePrimer code. The Figure S3 shown its validation curve.

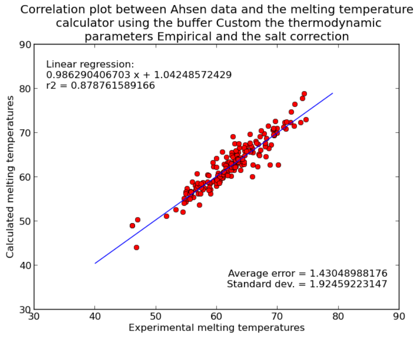

Figure S3: Validation curve of the empirical formula from [5]

S4 Experimental validation

We designed 96 primers using the default parameters of PrecisePrimes, except for the optimal melting temperature that was set at 60 °C for higher specificity of the primer on the genomic target, using the algorithm for NEB Q5 polymerase buffer. We amplified 61 different coding sequence fragments from yeast genomic extract (we reused the forward or reverse primer for some of them). On the first try we got 59 amplicons showing the correct size on agarose gel (Figure S4-a). The amplicon Flo1p-2 shows multiple band on gel because the primers turned to match several time on the gene due to the presence of repeated motifs.

We repeated the PCR for the two amplicons that did not worked in the first place, in order to check for pipeting errors or any other factor that may have affected the reaction. On the exact same conditions, the two PCR gave a band at the expect size on the gel (Figure S4-b).

Overall, all primers we designed successfully amplified their target sequence.

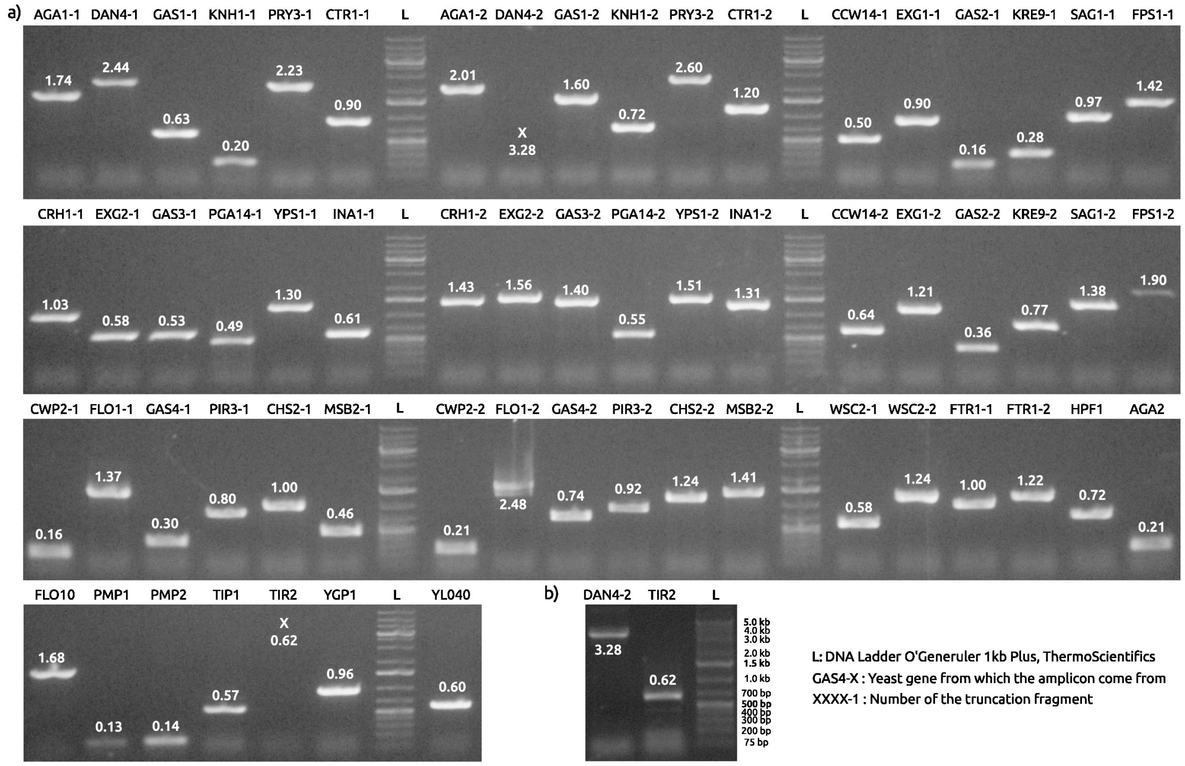

Figure S4: Agarose gel showing the amplicons after the PCR. The numbers in white corresponds to the theoretic size of the amplicon. a) PCR using Q5 DNA polymerase (NEB), default buffer and condition, b) We repeated the amplification of the two genes that didn't worked on the first round in the exact same conditions. The amplicon size is correct showing that the failure of the first PCR was due to experimental mistake and not to the primer design.

The primers used to amplify these genes are shown in Table S1.

Table S1: Primers used for the 61 PRC amplifications from yeast genomic DNA.

Primer Name	Sequence	Tm
AGA1-1-Fw	gaggtctctcttctGGAACTACAACCGTGGTATC	60.30
AGA1-2-Fw	gaggtctctcttctGGGGTTGTTACAACTACAGTTTC	61.43
AGA1-2-Rv	gcggtctctagtcTTAACTGAAAATTACATTGCAAGCAAC	60.69
CCW14-1-Fw	gaggtctctcttctGCTGCTTATTCTGCTTTCAAG	59.70
CCW14-2-Fw	gaggtctctcttctGCTTGTTTATTGGCCTGTG	59.53
CCW14-2-Rv	gcggtctctagtcTTAGATTAACATAGCGGCAACAG	60.48
CRH1-1-Fw	gaggtctctcttctGGTGTCGACACTCCTAC	59.20
CRH1-2-Fw	gaggtctctcttctGCAGCTTCTAGCACTGC	61.32
CRH1-2-Rv	gcggtctctagtcTTATAGCAAGGCTAGTAAAGCTAC	59.50
CWP2-1-Fw	gaggtctctcttctGCTACCACCGAAGCTAC	60.00
CWP2-2-Fw	gaggtctctcttctGCTGCCGCCATTTC	58.50
CWP2-2-Rv	gcggtctctagtcTTATAACAACATAGCAGCAGCAG	60.74
DAN4-1-Fw	gaggtctctcttctGCAACTACTACTACTACGAGC	59.64
DAN4-2-Fw	gaggtctctcttctGCAGCAGCCGTTTTTG	60.00
DAN4-2-Rv	gcggtctctagtcCTATAGCAGTAATAAAGCGACAAC	58.89
EXG1-1-Fw	gaggtctctcttctGGCCTACAGGAATCTTACC	59.65
EXG1-2-Fw	gaggtctctcttctGGTTCCCTCGGAGAAC	59.34
EXG1-2-Rv	gcggtctctagtcTTAGTTAGAAATTGTGCCACATTG	59.13
EXG2-1-Fw	gaggtctctcttctGCAGTAGTCGGAGAATGG	59.94
EXG2-2-Fw	gaggtctctcttctGCCTCCTACTATGCAAACG	60.78

Table S1 – continued

Primer Name	Sequence	Tm
EXG2-2-Rv	gcggtctctagtcTTAAAGAGAAGCGCAAAGAGC	61.04
FLO1-1-Fw	gaggtctctcttctGCCATCTCATCCAGTTTGTC	61.23
FLO1-2-Fw	gaggtctctcttctATCATTGTCATCAGAACACCAAC	60.92
FLO1-2-Rv	gcggtctctagtcTTAAATAATTGCCAGCAATAAGGAC	59.27
GAS1-1-Fw	gaggtctctcttctGGTAAGTACTGGTCCGC	59.64
GAS1-2-Fw	gaggtctctcttctGGTAATAAGTTTTTCTACTCCAACAAC	59.78
GAS1-2-Rv	gcggtctctagtcTTAAACCAAAGCAAAACCGAC	59.34
GAS2-1-Fw	gaggtctctcttctGGCGATTATTCAAAATCAAATCCATC	60.73
GAS2-2-Fw	gaggtctctcttctGGTGAATTCTCAGATTGTTCTG	58.84
GAS2-2-Rv	gcggtctctagtcTTATAGAAGAATTCCAGCAGCTATC	59.77
GAS3-1-Fw	gaggtctctcttctGGTTTGGTTAAGCTTGATGATAG	59.16
GAS3-2-Fw	gaggtctctcttctGGTGGGTCGTCTGG	59.07
GAS3-2-Rv	gcggtctctagtcTCAGAGTAGAGCAGAAATCAG	58.68
GAS4-1-Fw	gaggtctctcttctGGAAGTTCTTTGATAAAAAAAGGCG	60.79
GAS4-2-Fw	gaggtctctcttctAGCGTTGATTTTTACGGAGTTAAC	61.39
GAS4-2-Rv	gcggtctctagtcCTAAAAAAATAAACTAAAAGTAAAAAAAAGCAAACAC	59.93
KNH1-1-Fw	gaggtctctcttctAGTGAGGTAACGTATTACTACAC	58.87
KNH1-2-Fw	gaggtctctcttctTCAGTTTATGATTTATCTGGTACAAGTC	60.33
KNH1-2-Rv	gcggtctctagtcTTATCTGTGCCTCAAAGCATTAAC	61.09
KRE9-1-Fw	gaggtctctcttctGGTACGTCGCGTTTCG	61.28
KRE9-2-Fw	gaggtctctcttctGGAGATGTGAATATTGTTTCCCC	61.24
KRE9-2-Rv	gcggtctctagtcTCATACTTTTCTCATGTTGATTTTCC	59.12
PGA14-1-Fw	gaggtctctcttctTCTACTCATACCACTCACAAGTATG	61.21
PGA14-2-Fw	gaggtctctcttctGGTGAACATGATTTCACTACC	58.61
PGA14-2-Rv	gcggtctctagtcTTATAATAGTAATAAGGCACCGGC	59.86
PIR3-1-Fw	gaggtctctcttctGTCGCCTCATCTAAAGCAAAG	61.56
PIR3-2-Fw	gaggtctctcttctGCCTATGCTCCAAAGGAC	60.66
PIR3-2-Rv	gcggtctctagtcTCAACAGTCAATTAAATCTATAGCTTGC	61.17
PRY3-1-Fw	gaggtctctcttctGTGTGCTCCTACAACCC	60.27
PRY3-2-Fw	gaggtctctcttctGCACAAACCACGTTTCC	59.48
PRY3-2-Rv	gcggtctctagtcCTAGAAGGCGAACAGAACAG	60.51
SAG1-1-Fw	gaggtctctcttctGGTACAGCTAGCGCC	59.93
SAG1-2-Fw	gaggtctctcttctGGTTCTTTTGAAAGTTATCATTTGGG	60.56
SAG1-2-Rv	gcggtctctagtcTTAGAATAGCAGGTACGACAAAAG	60.06
YPS1-1-Fw	gaggtctctcttctGGCGGATCTTTGATTAATGATATAAAC	59.86
YPS1-2-Fw	gaggtctctcttctGCTGACGGTTATGAAGAAATTATAATTAC	60.06
YPS1-2-Rv	gcggtctctagtcTCAGATGAATGCAAAAAGAAGAG	58.67
CHS2-1-Fw	gaggtctctcttctTCTGTTGTACGCAAGTTCTTTC	60.79
CHS2-2-Fw	gaggtctctcttctGTTTTCACTGCAAATATGTACTTGG	60.36
CHS2-2-Rv	gcggtctctagtcTTAGCCCTTTTTGTGGAAAAC	58.87
CTR1-1-Fw	gaggtctctcttctTCATCGTCTGATAACAGTAGC	59.00
CTR1-2-Fw	gaggtctctcttctAGCAGCATGAATATGGACG	60.10
CTR1-2-Rv	gcggtctctagtcTTAGTTATGAGTGAATTTTTCGGC	58.91
FPS1-1-Fw	gaggtctctcttctGGCAGGACTACTGGTG	59.36
FPS1-2-Fw	gaggtctctcttctTCTTCATCACAACCTTCACATC	60.20
FPS1-2-Rv	gcggtctctagtcTCATGTTACCTTCTTAGCATTACC	59.94
INA1-1-Fw	gaggtctctcttctAGCAAGTCACACGCTG	60.05
INA1-2-Fw	gaggtctctcttctTCTAGTCTAAAAACCAGTCTTGC	59.83
INA1-2-Rv	gcggtctctagtcTTACCTTGCAAAGTTTTTTTCAGC	60.41
MSB2-1-Fw	gaggtctctcttctTCTTCCGGAAATTCCTATCAAG	59.56
MSB2-2-Fw	gaggtctctcttctGGAGCGCTTTCTGAAAGTAG	61.11
MSB2-2-Rv	gcggtctctagtcTCAAACTTCGTTCCAACCC	60.36
WSC2-1-Fw	gaggtctctcttctTCGTCCAAGAGCAAAGG	59.34
WSC2-2-Fw	gaggtctctcttctTCGAGCAAATGTGATGTTTCG	61.29
WSC2-2-Rv	gcggtctctagtcTCAGCGCAAAGAACTATTATTGG	60.96

Table S1 – continued

Primer Name	Sequence	Tm
FTR1-1-Rv	gcggtctctagtcTTATCCTGGGTTCAAGTGTTTC	60.22
FTR1-2-Rv	gcggtctctagtcTCAAAGAGAGTCGGCTTTAAC	59.94
FTR1-1-Fw	gaggtctctcttctATGCCTAACAAAGTGTTTAACG	58.86
HPF1-1-Fw	gaggtctctcttctGGATGTCATGTAACTACGTCTAC	60.07
HPF1-1-Rv	gcggtctctagtcTTAGTTAAAGAAAGCAAGAACGAAAATAC	59.91
AGA2-1-Fw	gaggtctctcttctGCACAGGAACTGACAACTATATG	61.08
AGA2-1-Rv	gcggtctctagtcTCAAAAAACATACTGTGTGTTTATGG	59.66
FLO10-1-Fw	gaggtctctcttctGCTACTTCATTTACCGCTTC	58.65
FLO10-1-Rv	gcggtctctagtcTTAAACGATTGCCAGTAATAGGG	60.27
PMP1-Fw	gaggtctctcttctATGACTTTACCAGGTGGTG	59.30
PMP1-Rv	gcggtctctagtcTTAGAATCTTTGCAATCCTCTTTGTC	61.09
PMP2-Fw	gaggtctctcttctATGTTGATGAGCACGTTACC	60.41
PMP2-Rv	gcggtctctagtcTTAGAATCTTTGTAAACCTCTTTGTCTAG	60.38
TIP1-1-Fw	gaggtctctcttctGCCGCCGAAACTGC	62.30
TIP1-1-Rv	gcggtctctagtcTTATAACAATAAAGCAGCTGCAC	58.99
TIR2-1-Fw	gaggtctctcttctGCTGAAGATTCTTCATCTGG	58.13
TIR2-1-Rv	gcggtctctagtcTTATAATAACATGGCGGCAGC	60.82
YGP1-1-Fw	gaggtctctcttctAGTAAGAATATCAACTCCACTTTGC	60.53
YGP1-1-Rv	gcggtctctagtcTCATGGGAAAATGCTTTCCAG	60.97
YL040-1-Fw	gaggtctctcttctGCCAAAACTTTAACGTCTAGTTC	59.68
YL040-1-Rv	gcggtctctagtcTTAAAGGGCAACTGCAAAG	58.36

S5 Primer design algorithm

PrecisePrimer's routine and melting temperature calculator has been developped on Python 2.7. This FASTA file parsing is taken from BioPython library. The graphs has been generated using matplotlib pyplot and numpy. The webserver is developped in html, css, javascript and PHP and hosted on a Apache 2.2.3 server.

The primer design algorithm is working as follow:

```
main(sequence, Tm_parameters):

Check inputs and retreive sequences

Retreive and check Tm calculations parameters

For each sequence in sequences
     internal_primer, Tm = design_primer(sequence)
     FW_primer = cloning_extension_FW + internal_primer
     store FW_primer and Tm in output FASTA and CSV file

     RC_sequence = Sequence_Reverse_Complement(sequence)
     RC_cloning_extension_RV = Sequence_Reverse_Complement(cloning_extension_RV)

     internal_primer, Tm = design_primer(RC_sequence)
     RV_primer = cloning_extension_FW + internal_primer
     store RV_primer and Tm in output FASTA and CSV file

END;
```

```
design_primer(sequence):

For i from 4 to len(sequence)
     Tm = calculate_melting_temperature(sequence[0:i])
     if Tm > Tm_opt - tolerance -> break

While Tm < Tm_opt + tolerance
     primer_list <- sequence[0:i], Tm
     i++

primer_list = sort_by_(Tm-Tm_opt)(primer_list)

If GC_clamp = True
     for sequence in primer_list
         if sequence[-1] = G or C
              return sequence, Tm
Else
     return primer_list_first_sequence, primer_list_first_Tm

END;
```

References

[1] K. J. Breslauer, R. Frank, H. Blöcker, and L. A. Marky, "Predicting DNA duplex stability from the base sequence," *PNAS*, vol. 83, no. june, pp. 3746–3750, 1986.

[2] J. J. SantaLucia, "A unified view of polymer, dumbbell, and oligonucleotide DNA nearest-neighbor thermodynamics," *Proc Natl Acad Sci U S A*, vol. 95, no. February, pp. 1460–1465, 1998.

[3] C. Schildkraut, "Dependence of the melting temperature of DNA on salt concentration.," *Biopolymers*, vol. 3, pp. 195–208, Jan. 1965.

[4] R. Owczarzy, I. Dunietz, M. A. Behlke, I. M. Klotz, and J. A. Walder, "Thermodynamic treatment of oligonucleotide duplex-simplex equilibria.," *Proceedings of the National Academy of Sciences of the United States of America*, vol. 100, pp. 14840–5, Dec. 2003.

[5] C. T. Wittwer, E. Schu, and N. Von Ahsen, "Oligonucleotide Melting Temperatures under PCR Conditions: Nearest-Neighbor Corrections for Mg," *Clinical chemistry*, vol. 47, no. 11, pp. 1956–1961, 2001.

[6] H. Musielski, W. Mann, R. Laue, and S. Michel, "Influence of dimethylsulfoxide on transcription by bacteriophage T3-induced RNA polymerase," *Zeitschrift für allgemeine Mikrobiologie*, vol. 21, pp. 447–456, Jan. 2007.
